# Tris(3-chloro­pentane-2,4-dionato-κ^2^ *O*,*O*′)aluminium

**DOI:** 10.1107/S1600536812023203

**Published:** 2012-05-26

**Authors:** Franc Perdih

**Affiliations:** a Faculty of Chemistry and Chemical Technology, University of Ljubljana, Aškerčeva 5, PO Box 537, SI-1000 Ljubljana, Slovenia; b CO EN–FIST, Dunajska 156, SI-1000 Ljubljana, Slovenia

## Abstract

In the title compound, [Al(C_5_H_6_ClO_2_)_3_], the Al^III^ cation is situated on a twofold rotation axis and is coordinated by six O atoms from three 3-chloro­pentane-2,4-dionate ligands in an octa­hedral environment. Al—O bond lengths are in the range 1.8741 (14)–1.8772 (14) Å. In the crystal, mol­ecules are linked *via* C—H⋯Cl contacts.

## Related literature

For applications of metal complexes with β-diketonate ligands, see: Bray *et al.* (2007 ▶); Garibay *et al.* (2009 ▶); Lichtenberger *et al.* (2010 ▶); Perdih (2011 ▶); Vreshch *et al.* (2004 ▶); Wu & Wang (2009 ▶). For related structures, see: Hon & Pfluger (1973 ▶); Perdih (2012 ▶).
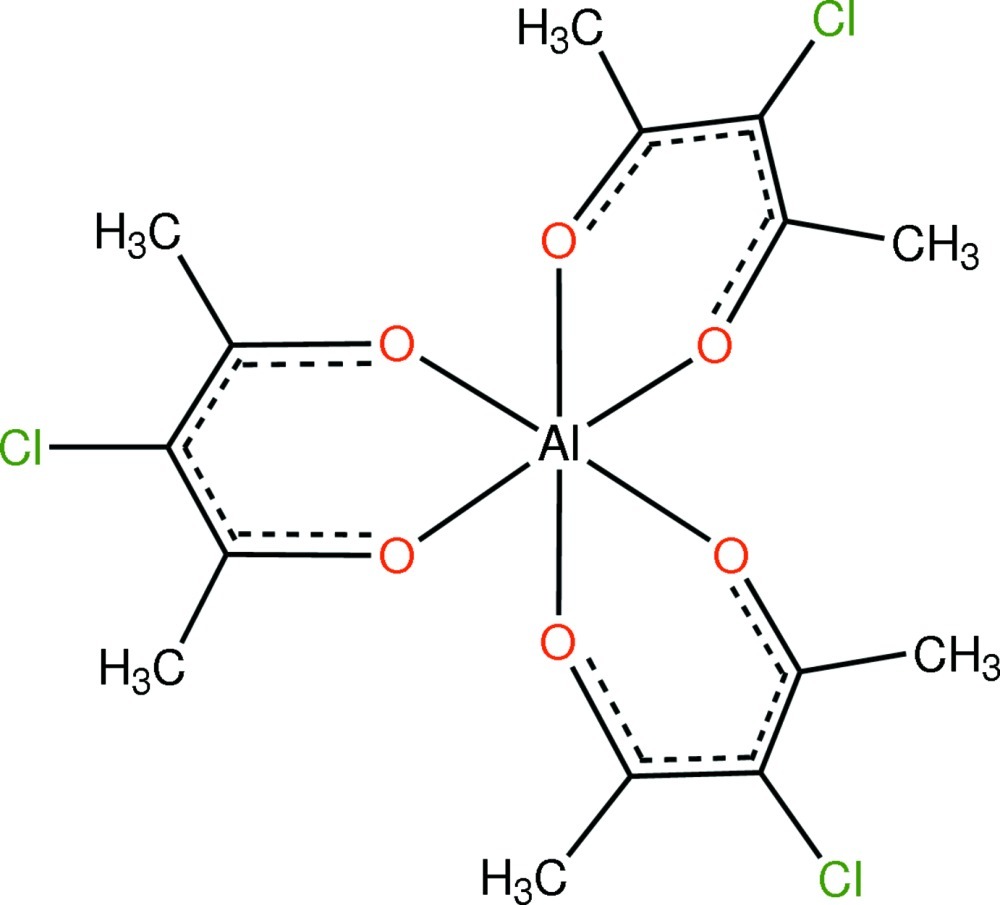


## Experimental

### 

#### Crystal data

[Al(C_5_H_6_ClO_2_)_3_]
*M* *_r_* = 427.62
Monoclinic, 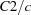

*a* = 12.8790 (3) Å
*b* = 9.9086 (2) Å
*c* = 15.5311 (4) Å
β = 106.368 (2)°
*V* = 1901.64 (8) Å^3^
*Z* = 4
Mo *K*α radiation
μ = 0.56 mm^−1^
*T* = 293 K
0.33 × 0.25 × 0.08 mm

#### Data collection

Nonius KappaCCD area-detector diffractometer
Absorption correction: multi-scan (*SCALEPACK*; Otwinowski & Minor, 1997 ▶) *T*_min_ = 0.838, *T*_max_ = 0.957
3916 measured reflections
2156 independent reflections
1759 reflections with *I* > 2σ(*I*)
*R*_int_ = 0.014

#### Refinement

*R*[*F* ^2^ > 2σ(*F* ^2^)] = 0.042
*wR*(*F* ^2^) = 0.125
*S* = 1.05
2156 reflections
118 parameters
H-atom parameters constrained
Δρ_max_ = 0.33 e Å^−3^
Δρ_min_ = −0.34 e Å^−3^

### 

Data collection: *COLLECT* (Hooft, 1998 ▶); cell refinement: *DENZO-SMN* (Otwinowski & Minor, 1997 ▶); data reduction: *DENZO-SMN*; program(s) used to solve structure: *SIR97* (Altomare *et al.*, 1999 ▶); program(s) used to refine structure: *SHELXL97* (Sheldrick, 2008 ▶); molecular graphics: *ORTEP-3 for Windows* (Farrugia, 1997 ▶); software used to prepare material for publication: *WinGX* (Farrugia, 1999 ▶) and *publCIF* (Westrip, 2010 ▶).

## Supplementary Material

Crystal structure: contains datablock(s) I, global. DOI: 10.1107/S1600536812023203/im2374sup1.cif

Structure factors: contains datablock(s) I. DOI: 10.1107/S1600536812023203/im2374Isup2.hkl

Additional supplementary materials:  crystallographic information; 3D view; checkCIF report

## Figures and Tables

**Table 1:** Hydrogen-bond geometry (Å, °)

*D*—H⋯*A*	*D*—H	H⋯*A*	*D*⋯*A*	*D*—H⋯*A*
C6—H6*A*⋯Cl1^i^	0.96	2.94	3.796 (2)	149

Symmetry code: (i) 
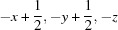
.

**Fig. 1.**
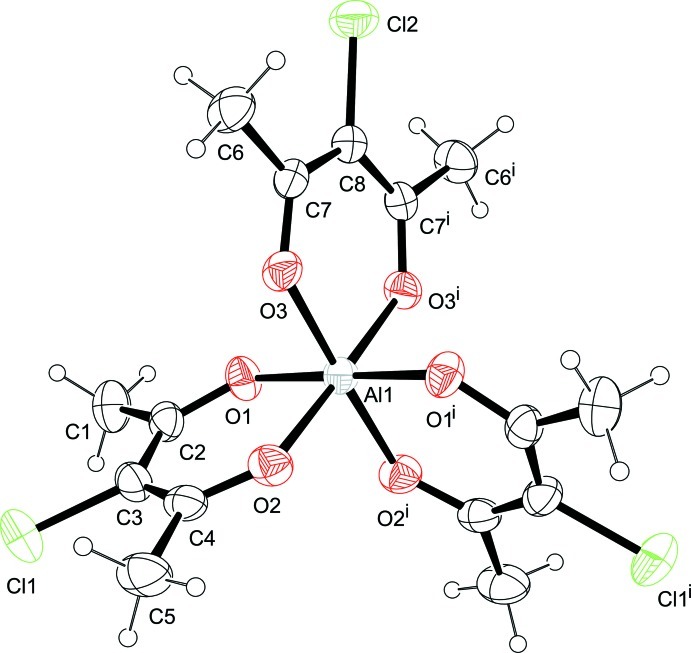
Molecular structure of the title complex showing displacement ellipsoids at the 30% probability level. Symmetry code: i = –x + 1, y, –z + 1/2.

**Fig. 2.**
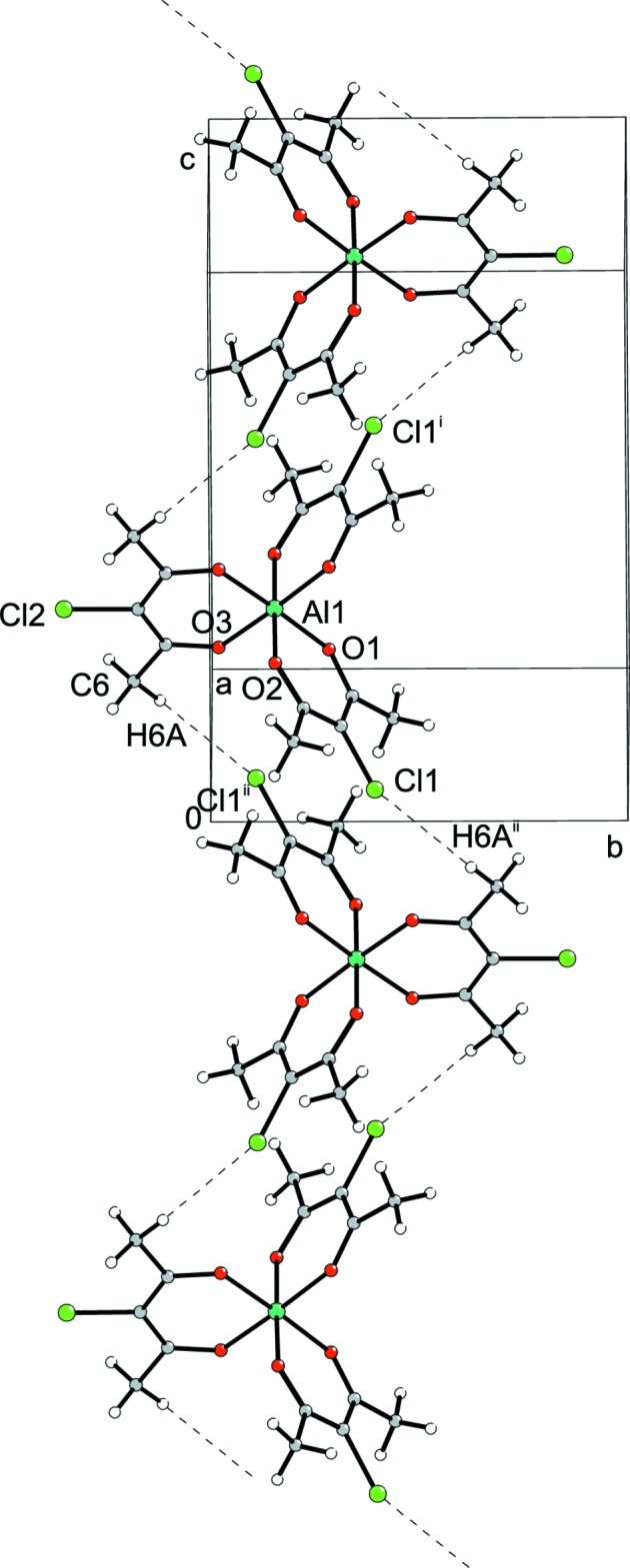
1D infinte chain with dashed lines indicating intermolecular C6—H6A···Cl1 hydrogen bonding. For the sake of clarity, H atoms not involved in the motif shown have been omitted. Symmetry code: ii = –x + 1/2, –y + 1/2, –z.

## supplementary crystallographic information

### Comment

β-Diketonates have been proven to be versatile ligands for various metal ions. They can be easily derivatized, thus modifying the electronic and steric nature of these ligands to design suitable structure/function relationships (Bray *et al.*, 2007; Garibay *et al.*, 2009; Perdih, 2011). β-Diketonate compounds of aluminium have received great attention due to the promise of the construction of cages (Vreshch *et al.*, 2004; Wu & Wang, 2009). Besides that, aluminium β-diketonates and malonates can be good precursors in metal-organic chemical vapour deposition (MOCVD) (Bray *et al.*, 2007; Garibay *et al.*, 2009; Lichtenberger *et al.*, 2010).

In the title molecule (Fig. 1), the aluminium(III) cation is situated on a twofold axis, and is surrounded by six O atoms from three 3-chloropentane-2,4-dionate ligands in a octahedral environment. The geometry around aluminium is close to the orthogonallity as can be seen from the angles. The Al—O bond lengths are in the range 1.8741 (14)–1.8772 (14) Å and are similar as for example in Al(acac)_3_ (Hon & Pfluger, 1973). The displacement of the metal atom is best described by a bending of a chelate ligand about the "bite" atoms. The angles between the O—Al—O and the ligand chelate mean planes are 0.38° and 1.72°. For comparison these values are 0.78° and 12.68° in the isostructural iron(III) compound (Perdih, 2012). A 1-D framework is achieved due to intermolecular C6–H6A···Cl1 (–*x* + 1/2, –*y* + 1/2, –*z*) contacts (Fig. 2).

### Experimental

To a clear solution of Al_2_(SO_4_)_3_^.^18H_2_O (1 mmol, 0.67 g) in water (15 ml) a solution of 3-chloropentane-2,4-dione (6 mmol, 0.81 g) in methanol (5 ml) was added while stirring. Afterwards 1 *M* NaOH (6 ml) was slowly added and the resulting solution was stirred at 70°C for 15 minutes. After cooling to room temperature the light pink product was filtrated, washed with water (20 ml), and subsequently air-dried. Yield: 0.60 g, 70%. Crystals suitable for X-ray analysis were obtained by recrystallization from ethanol.

### Refinement

All H atoms were initially located in a difference Fourier maps and were subsequently treated as riding atoms in geometrically idealized positions, with C—H = 0.96 Å, and with *U*_iso_(H) = 1.5*U*_eq_(C).

### Crystal data

**Table:**

[Al(C_5_H_6_ClO_2_)_3_]	*F*(000) = 880
*M**_r_* = 427.62	*D*_x_ = 1.494 Mg m^−^^3^
Monoclinic, *C*2/*c*	Mo *K*α radiation, λ = 0.71073 Å
Hall symbol: -C 2yc	Cell parameters from 2269 reflections
*a* = 12.8790 (3) Å	θ = 2.6–27.5°
*b* = 9.9086 (2) Å	µ = 0.56 mm^−^^1^
*c* = 15.5311 (4) Å	*T* = 293 K
β = 106.368 (2)°	Plate, pink
*V* = 1901.64 (8) Å^3^	0.33 × 0.25 × 0.08 mm
*Z* = 4	

### Data collection

**Table:**

Nonius KappaCCD area-detector diffractometer	2156 independent reflections
Graphite monochromator	1759 reflections with *I* > 2σ(*I*)
Detector resolution: 0.055 pixels mm^-1^	*R*_int_ = 0.014
ω scans	θ_max_ = 27.5°, θ_min_ = 5.6°
Absorption correction: multi-scan (*SCALEPACK*; Otwinowski & Minor, 1997)	*h* = −16→16
*T*_min_ = 0.838, *T*_max_ = 0.957	*k* = −10→12
3916 measured reflections	*l* = −20→20

### Refinement

**Table:**

Refinement on *F*^2^	Primary atom site location: structure-invariant direct methods
Least-squares matrix: full	Secondary atom site location: difference Fourier map
*R*[*F*^2^ > 2σ(*F*^2^)] = 0.042	Hydrogen site location: inferred from neighbouring sites
*wR*(*F*^2^) = 0.125	H-atom parameters constrained
*S* = 1.05	*w* = 1/[σ^2^(*F*_o_^2^) + (0.0632*P*)^2^ + 1.1678*P*] where *P* = (*F*_o_^2^ + 2*F*_c_^2^)/3
2156 reflections	(Δ/σ)_max_ < 0.001
118 parameters	Δρ_max_ = 0.33 e Å^−^^3^
0 restraints	Δρ_min_ = −0.34 e Å^−^^3^

### Special details

**Table:**

Experimental. 211 frames in 5 sets of ω scans. Rotation/frame = 2.0 °. Crystal-detector distance = 25.00 mm. Measuring time = 55 s/°.
Geometry. All s.u.'s (except the s.u. in the dihedral angle between two l.s. planes) are estimated using the full covariance matrix. The cell s.u.'s are taken into account individually in the estimation of s.u.'s in distances, angles and torsion angles; correlations between s.u.'s in cell parameters are only used when they are defined by crystal symmetry. An approximate (isotropic) treatment of cell s.u.'s is used for estimating s.u.'s involving l.s. planes.
Refinement. Refinement of *F*^2^ against ALL reflections. The weighted *R*-factor *wR* and goodness of fit *S* are based on *F*^2^, conventional *R*-factors *R* are based on *F*, with *F* set to zero for negative *F*^2^. The threshold expression of *F*^2^ > 2σ(*F*^2^) is used only for calculating *R*-factors(gt) *etc*. and is not relevant to the choice of reflections for refinement. *R*-factors based on *F*^2^ are statistically about twice as large as those based on *F*, and *R*- factors based on ALL data will be even larger.

### Fractional atomic coordinates and isotropic or equivalent isotropic displacement parameters (Å^2^)

**Table:**

	*x*	*y*	*z*	*U*_iso_*/*U*_eq_	
Al1	0.5	0.15245 (8)	0.25	0.0454 (2)	
Cl1	0.36200 (7)	0.39260 (8)	−0.04272 (5)	0.0995 (3)	
Cl2	0.5	−0.35153 (8)	0.25	0.0799 (3)	
O1	0.39394 (11)	0.28432 (14)	0.20496 (10)	0.0575 (4)	
O2	0.53620 (11)	0.15368 (15)	0.14118 (9)	0.0565 (4)	
O3	0.39461 (10)	0.01885 (13)	0.20911 (9)	0.0527 (3)	
C1	0.2712 (2)	0.4373 (3)	0.1141 (2)	0.0824 (8)	
H1A	0.2095	0.4025	0.0694	0.124*	
H1B	0.292	0.5223	0.0945	0.124*	
H1C	0.2532	0.4496	0.1695	0.124*	
C2	0.36336 (15)	0.33939 (18)	0.12819 (15)	0.0558 (5)	
C3	0.41130 (17)	0.3110 (2)	0.06059 (14)	0.0587 (5)	
C4	0.49653 (15)	0.2202 (2)	0.06969 (12)	0.0529 (4)	
C5	0.5460 (2)	0.1956 (3)	−0.00518 (15)	0.0756 (7)	
H5A	0.6094	0.1406	0.016	0.113*	
H5B	0.5656	0.2803	−0.0262	0.113*	
H5C	0.4947	0.1502	−0.0534	0.113*	
C6	0.29806 (17)	−0.1843 (2)	0.17442 (17)	0.0671 (6)	
H6A	0.2393	−0.1214	0.156	0.101*	
H6B	0.2852	−0.2439	0.2191	0.101*	
H6C	0.3034	−0.2359	0.1235	0.101*	
C7	0.40167 (15)	−0.10871 (18)	0.21301 (11)	0.0468 (4)	
C8	0.5	−0.1744 (3)	0.25	0.0493 (6)	

### Atomic displacement parameters (Å^2^)

**Table:**

	*U*^11^	*U*^22^	*U*^33^	*U*^12^	*U*^13^	*U*^23^
Al1	0.0412 (4)	0.0466 (4)	0.0457 (4)	0	0.0078 (3)	0
Cl1	0.1147 (6)	0.0930 (5)	0.0719 (4)	0.0188 (4)	−0.0043 (4)	0.0304 (3)
Cl2	0.0779 (6)	0.0479 (4)	0.0966 (6)	0	−0.0039 (4)	0
O1	0.0517 (7)	0.0531 (8)	0.0671 (8)	0.0097 (6)	0.0155 (6)	0.0005 (6)
O2	0.0512 (7)	0.0686 (8)	0.0482 (7)	0.0139 (6)	0.0116 (5)	0.0076 (6)
O3	0.0422 (6)	0.0522 (7)	0.0565 (7)	0.0005 (5)	0.0021 (5)	−0.0059 (6)
C1	0.0621 (13)	0.0577 (12)	0.114 (2)	0.0167 (11)	0.0029 (13)	−0.0025 (13)
C2	0.0430 (9)	0.0406 (9)	0.0739 (13)	−0.0008 (7)	0.0002 (8)	−0.0001 (8)
C3	0.0546 (11)	0.0536 (10)	0.0573 (11)	−0.0005 (9)	−0.0015 (8)	0.0105 (9)
C4	0.0461 (9)	0.0589 (10)	0.0483 (9)	−0.0071 (8)	0.0043 (7)	0.0034 (8)
C5	0.0700 (14)	0.1046 (19)	0.0528 (11)	0.0003 (14)	0.0182 (10)	0.0051 (12)
C6	0.0496 (11)	0.0674 (13)	0.0775 (14)	−0.0105 (10)	0.0066 (10)	−0.0102 (11)
C7	0.0457 (9)	0.0526 (10)	0.0400 (8)	−0.0046 (7)	0.0088 (7)	−0.0045 (7)
C8	0.0512 (14)	0.0484 (13)	0.0443 (12)	0	0.0072 (10)	0

### Geometric parameters (Å, º)

**Table:**

Al1—O3	1.8741 (14)	C1—H1C	0.96
Al1—O3^i^	1.8741 (14)	C2—C3	1.389 (3)
Al1—O2^i^	1.8756 (13)	C3—C4	1.395 (3)
Al1—O2	1.8756 (13)	C4—C5	1.495 (3)
Al1—O1^i^	1.8772 (14)	C5—H5A	0.96
Al1—O1	1.8772 (14)	C5—H5B	0.96
Cl1—C3	1.748 (2)	C5—H5C	0.96
Cl2—C8	1.755 (3)	C6—C7	1.500 (3)
O1—C2	1.269 (3)	C6—H6A	0.96
O2—C4	1.268 (2)	C6—H6B	0.96
O3—C7	1.267 (2)	C6—H6C	0.96
C1—C2	1.500 (3)	C7—C8	1.396 (2)
C1—H1A	0.96	C8—C7^i^	1.396 (2)
C1—H1B	0.96		
			
O3—Al1—O3^i^	90.12 (8)	C3—C2—C1	121.5 (2)
O3—Al1—O2^i^	88.26 (6)	C2—C3—C4	123.97 (18)
O3^i^—Al1—O2^i^	92.26 (6)	C2—C3—Cl1	118.44 (16)
O3—Al1—O2	92.26 (6)	C4—C3—Cl1	117.59 (17)
O3^i^—Al1—O2	88.26 (6)	O2—C4—C3	122.35 (18)
O2^i^—Al1—O2	179.26 (10)	O2—C4—C5	116.20 (19)
O3—Al1—O1^i^	178.02 (6)	C3—C4—C5	121.44 (19)
O3^i^—Al1—O1^i^	89.08 (6)	C4—C5—H5A	109.5
O2^i^—Al1—O1^i^	89.96 (6)	C4—C5—H5B	109.5
O2—Al1—O1^i^	89.53 (7)	H5A—C5—H5B	109.5
O3—Al1—O1	89.08 (6)	C4—C5—H5C	109.5
O3^i^—Al1—O1	178.02 (6)	H5A—C5—H5C	109.5
O2^i^—Al1—O1	89.53 (7)	H5B—C5—H5C	109.5
O2—Al1—O1	89.96 (6)	C7—C6—H6A	109.5
O1^i^—Al1—O1	91.78 (9)	C7—C6—H6B	109.5
C2—O1—Al1	130.55 (13)	H6A—C6—H6B	109.5
C4—O2—Al1	130.63 (13)	C7—C6—H6C	109.5
C7—O3—Al1	130.76 (12)	H6A—C6—H6C	109.5
C2—C1—H1A	109.5	H6B—C6—H6C	109.5
C2—C1—H1B	109.5	O3—C7—C8	121.98 (17)
H1A—C1—H1B	109.5	O3—C7—C6	115.78 (17)
C2—C1—H1C	109.5	C8—C7—C6	122.24 (19)
H1A—C1—H1C	109.5	C7^i^—C8—C7	124.4 (2)
H1B—C1—H1C	109.5	C7^i^—C8—Cl2	117.82 (12)
O1—C2—C3	122.47 (17)	C7—C8—Cl2	117.82 (12)
O1—C2—C1	116.0 (2)		
			
O3—Al1—O1—C2	−89.43 (17)	C1—C2—C3—C4	−179.5 (2)
O2^i^—Al1—O1—C2	−177.70 (17)	O1—C2—C3—Cl1	179.76 (15)
O2—Al1—O1—C2	2.83 (18)	C1—C2—C3—Cl1	0.1 (3)
O1^i^—Al1—O1—C2	92.36 (17)	Al1—O2—C4—C3	−0.2 (3)
O3—Al1—O2—C4	87.74 (18)	Al1—O2—C4—C5	179.99 (15)
O3^i^—Al1—O2—C4	177.79 (18)	C2—C3—C4—O2	1.3 (3)
O1^i^—Al1—O2—C4	−93.12 (18)	Cl1—C3—C4—O2	−178.31 (15)
O1—Al1—O2—C4	−1.34 (18)	C2—C3—C4—C5	−179.0 (2)
O3^i^—Al1—O3—C7	1.05 (13)	Cl1—C3—C4—C5	1.4 (3)
O2^i^—Al1—O3—C7	−91.21 (17)	Al1—O3—C7—C8	−2.0 (3)
O2—Al1—O3—C7	89.32 (16)	Al1—O3—C7—C6	178.65 (13)
O1—Al1—O3—C7	179.24 (16)	O3—C7—C8—C7^i^	1.01 (12)
Al1—O1—C2—C3	−2.7 (3)	C6—C7—C8—C7^i^	−179.72 (19)
Al1—O1—C2—C1	176.98 (15)	O3—C7—C8—Cl2	−178.99 (12)
O1—C2—C3—C4	0.2 (3)	C6—C7—C8—Cl2	0.28 (19)

Symmetry code: (i) −*x*+1, *y*, −*z*+1/2.

### Hydrogen-bond geometry (Å, º)

**Table:**

*D*—H···*A*	*D*—H	H···*A*	*D*···*A*	*D*—H···*A*
C6—H6*A*···Cl1^ii^	0.96	2.94	3.796 (2)	149

Symmetry code: (ii) −*x*+1/2, −*y*+1/2, −*z*.
